# Therapeutic potential of arginine deprivation therapy for gliomas: a systematic review of the existing literature

**DOI:** 10.3389/fphar.2024.1446725

**Published:** 2024-08-22

**Authors:** Chen Yuxiao, Wang Jiachen, Lan Yanjie, Li Shenglan, Wang Yuji, Li Wenbin

**Affiliations:** ^1^ Department of Neuro-Oncology, Beijing Tiantan Hospital, Capital Medical University, Beijing, China; ^2^ Xuanwu Hospital (The First Clinical College of Capital Medical University), Beijing, China; ^3^ Department of Medicinal Chemistry, College of Pharmaceutical Sciences, Capital Medical University, Beijing, China

**Keywords:** glioma, arginine deprivation therapy, tumor metabolism, combination therapy, ADI-PEG20, BET-100

## Abstract

**Background:**

Arginine deprivation therapy (ADT) hinders glioma cells’ access to nutrients by reducing peripheral blood arginine, showing great efficacy in various studies, which suggests it as a potentially promising treatment for glioma. The aim of this systematic review was to explore the mechanism of ADT for gliomas, the therapeutic effect based on existing research, and possible combination therapies.

**Methods:**

We performed a systematic literature review of PubMed, ScienceDirect and Web of Science databases according to PRISMA guidelines, searching for articles on the efficacy of ADT in glioma.

**Results:**

We identified 17 studies among 786 search results, among which ADT therapy mainly based on Arginine free condition, Arginine Deiminase and Arginase, including three completed clinical trials. ADT therapy has shown promising results *in vivo* and *in vitro*, with its safety confirmed in clinical trials. In the early phase of treatment, glioblastoma (GBM) cells develop protective mechanisms of stress and autophagy, which eventually evolve into caspase dependent apoptosis or senescence, respectively. The immunosuppressive microenvironment is also altered by arginine depletion, such as the transformation of microglia into a pro-inflammatory phenotype and the activation of T-cells. Thus, ADT therapy demonstrates glioma-killing effect in the presence of a combination of mechanisms. In combination with various conventional therapies and investigational drugs such as radiotherapy, temozolomide (TMZ), cyclin-dependent kinase inhibitors (CDK) inhibitors and autophagy inducers, ADT therapy has been shown to be more effective. However, the phenomenon of drug resistance due to re-expression of ASS1 rather than stem cell remains to be investigated.

**Conclusion:**

Despite the paucity of studies in the literature, the available data demonstrate the therapeutic potential of arginine deprivation therapy for glioma and encourage further research, especially the exploration of its combination therapies and the extrapolation of what we know about the effects and mechanisms of ADT from other tumors to glioma.

## 1 Introduction

Gliomas, originating from the central nervous system’s (CNS) supportive glial cells, encompass a range of malignancies from benign to the highly aggressive glioblastomas (GBMs) (Davis, 2018). These tumors represent about 30% of primary brain tumors, with incidence ranging from 1.9 to 9.6 per 100,000 adults varying by age, sex, ethnicity, and geography (Śledzińska et al., 2021). The World Health Organization (WHO) grades gliomas into four categories, with grade I showing high survival rates and grade IV, the most severe, only having a 5-year survival rate of 6.8% (Ostrom et al., 2021). The standard glioma treatment protocol includes maximal safe surgical resection, followed by concurrent radiotherapy (RT) and TMZ, with an additional 6 months of TMZ. Despite Food and Drug Administration (FDA)-approved therapies like Tumor Treatment Fields (TTF) and Bevacizumab (BEV), limitations persist (Xu et al., 2020). Surgery carries the risk of damaging surrounding tissue and may not completely eradicate the tumor. RT can lead to radiation-induced neuro-logical deficits, while chemotherapy is associated with hematological side effects, including bone marrow suppression (Lee and Wee, 2022). The efficacy of immunotherapy in clinical trials has been limited (Xu et al., 2020). Despite its promise, TTF is limited by its high cost, lack of placebo-controlled clinical trials, the burden of long-term devices using on patients, and an incompletely understood mechanism of action (Rominiyi et al., 2021). BEV has shown limited impact on overall survival (OS) and is associated with drug resistance issues (Tamura et al., 2017). Given these limitations, the search for an effective treatment for glioma has been challenging. The search for novel therapeutic strategies is imperative and requires a broader exploration of potential breakthroughs.

The discovery of the Warburg effect has redirected our attention to the metabolic reprogramming of tumors. This process involves tumors adapting to their surroundings by modifying their cellular metabolism, a phenomenon recognized as a key characteristic of cancer (Hernández et al., 2021; Bi et al., 2020), This has broadened our understanding and introduced new avenues in cancer research (Caniglia et al., 2021). This metabolic plasticity includes a preference for glycolysis over oxidative phosphorylation, even in the presence of oxygen, leading to the production of lactic acid (Liberti and Locasale, 2016) and reactive oxygen species (ROS), which in turn support tumorigenesis (Martínez-Reyes and Chandel, 2021), and an increased requirement for endogenous or exogenous essential amino acids to support their rapid proliferation. Amino acid metabolism, particularly arginine, has been identified as a key factor in tumor biology, influencing the tumor microenvironment (TME) and immune cell phenotypes. Researchers have progressively explored the role of arginine metabolism on tumors, such as the conversion of arginine to urea and ornithine/citrulline and nitric oxide (NO), respectively, by arginase (Arg1)/nitric oxide synthase (NOS), which predisposes microglial cells to an anti-tumor M1/pro-tumor M2 phenotype (Bi et al., 2020; Bernhard et al., 2023).

**FIGURE 1:**
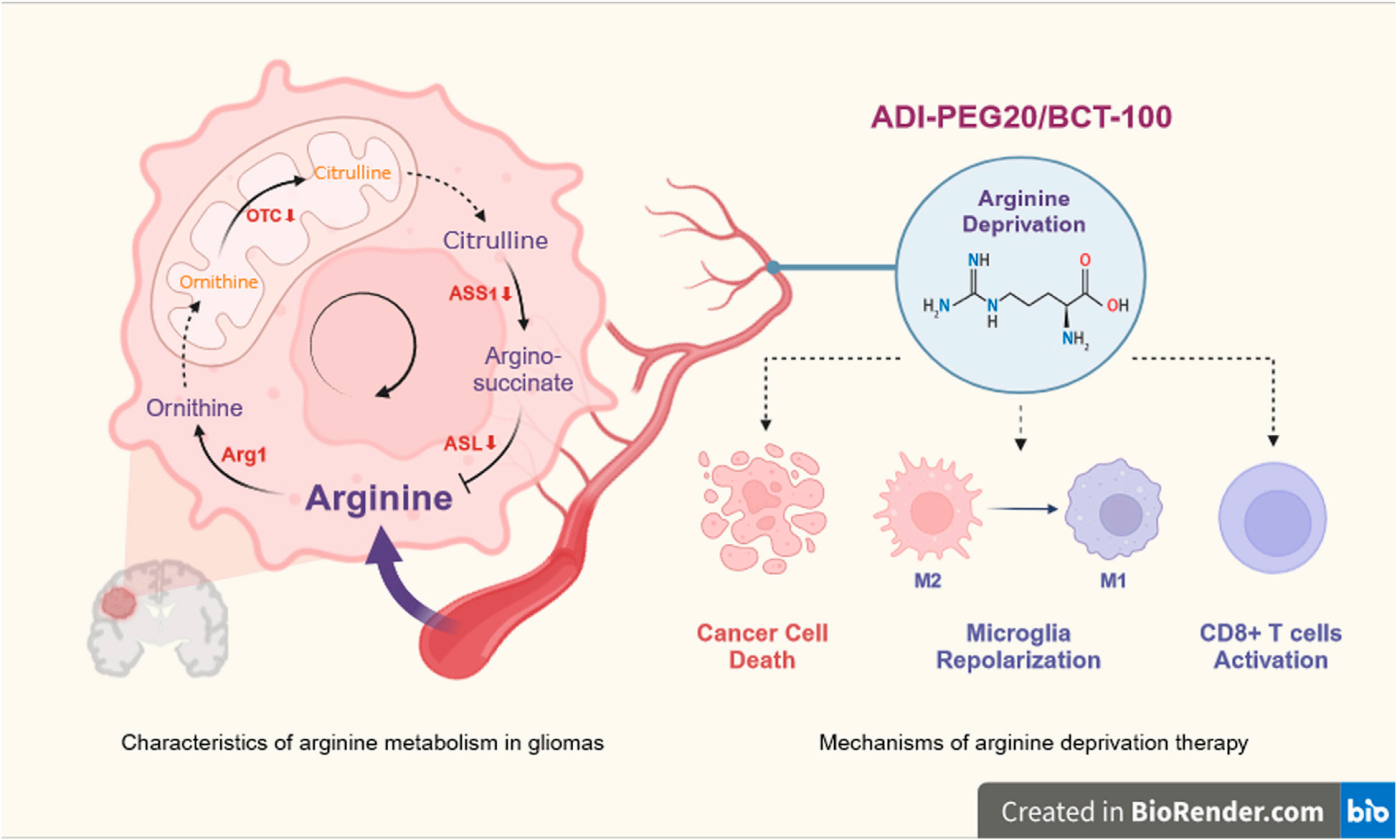
Overview of arginine deprivation therapy for glioma. Deficiency of ASL, ASS1 or OCT in GBM results in an inability to synthesis arginine endogenously and a high dependence on exogenous arginine. ADT therapy (ADI-PEG20 or BCT-100) deprives GBM of its nutritional source by lowering peripheral blood arginine, which acts as a killing agent for the cancer cells, and at the same time, will help the microglia to repolarize from M2 to M1 and CD8 +T cells to regain function. OTC, ornithine transcarboxylase; ASS1, argininosuccinate synthetase; ASL, argininosuccinatelyase, Arg1, arginase; ADI-PEG20, pegylated arginine deaminase; BCT-100, pegylated recombinant human arginase. Created with https://www.biorender.com/, accessed on 29 May 2024.

Arginine, a semi-essential amino acid, can be synthesized by normal cells from citrulline and aspartate in the urea cycle (Chen et al., 2021). Enzymes required for the endogenous synthesis of arginine by tumor cells include argininosuccinate synthase 1 (ASS1), argininosuccinatelyase (ASL) and ornithine carbamoyl transferase (OCT). ASS1 catalyzes the conversion of citrulline and aspartate to arginine succinate, which is later cleaved by ASL to L-arginine and fumarate, and arginine is further hydrolyzed by Arg1 to urea and ornithine (Huang et al., 2014). OCT catalyzes the formation of citrulline from ornithine and carbamoyl phosphate. However, these three enzymes are aberrantly expressed in some tumors, resulting in the inability of tumor cells to produce endogenous arginine, making the tumor highly dependent on exogenous arginine. This type of tumor is known as argi-nine-deficient tumors (Du et al., 2022), including gliomas (Zhang et al., 2022). Arginine deprivation therapy (ADT) has emerged as a potential therapeutic strategy to target this metabolic vulnerability (Figure 1).

The core mechanism of arginine deprivation therapy revolves around arginine deiminase (ADI) and arginase (Arg1), which work through different mechanisms to deplete arginine in the tumor microenvironment (Table 1). ADI, derived from *Streptococcus* pyogenes, and its pharmacological formulation pegylated arginine deiminase (ADI-PEG20) have shown efficacy linked to ASS1/ASL expression levels. In 2002, Ensor et al. (2002) published the first study linking ASS1 expression to the efficacy of ADI-PEG20 in melanoma and hepatocellular carcinoma cell lines, which showed remarkable results in early clinical trials and was granted orphan drug designation by the FDA for the treatment of malignant melanoma and hepatocellular carcinoma (HCC). Meanwhile, the main Arg1-based drug formulation is pegylated recombinant arginase (BCT-100), which is obtained through the production of His-tagged human liver arginase I in *Bacillus subtilis* (F and Cg, 2013; Pn et al., 2007). The investigators initially conjugated Mn2+ to improve stability; however, in serum, Mn2+ tends to be rapidly released from the protein, leading to enzyme inactivation and a half-life of only 4.8 h. To overcome this challenge, the replacement of Mn2+ by Co2+ was developed and the half-life of modified Arg1 was extended to 6.1 h (Fultang et al., 2016), due to the increased size of the molecule and reduced immunogenicity, preventing glomerular filtration loss (Mauldin et al., 2012; Tanios et al., 2013). The overall catalytic activity (kcat/KM) was increased by a factor of 10 under conditions close to human serum (pH = 7.4) (Stone et al., 2010). Both ADI and Arg1 are rapidly removed from the circulation by glomerular filtration, a process that is achieved within 30 min. The duration of plasma arginine elimination increased as the size of the PEG was increased from 5 kDa to 20 kDa, and further increases did not significantly prolong the time to plasma arginine depletion, making 20 kDa PEG optimal for ADI, with a prolongation of half-life that can be achieved by exceeding renal thresholds (Holtsberg et al., 2002). And further studies have shown that the half-life of ADI was significantly improved when ADI was covalently linked to 16 20 kDa PEG; the half-life was significantly improved and could be increased to 7 days with optimal activity at physiological pH (Fultang et al., 2016). In contrast, Arg1 was shown in a patent to be significantly covalently linked to 5,000 Da of mPEG-SPA (methoxypolyethylene glycol succinimidyl propionate) (CHENG et al., 2008). PEGylation of Arg1 was shown to increase its half-life to 3 days *in vivo* (Pn et al., 2007; Riess et al., 2018). Therefore, efficacy has been validated in clinical trials using the form of PEGylated ADI and Arg1, namely, ADI-PEG20 and BCT-100, as one of the combination drugs.

**TABLE 1:** Characteristics of arginine deprivation therapy based on arginine deaminase and arginase.

Characteristics	ADI			ArgⅠ	
Types	ADI-PEG20			BCT-100	HuArgI (Co)-PEG5000
Origin	*M. hominis*	*M. arginine*	*S. pyogenes*	Human	
Km	0.1–12 (7)	0.2	1.33 ± 0.12	2.914	
Optimal pH	6.0	6.0–7.5	6.5	8.5	7.5
Half-life	7 days (Ensor et al., 2002)			2–3 days	
PEG (Mw)	20,000			5,000	
Relative genes	ASS1/ASL			OTC	

Abbreviation: ADI, arginine deaminase; Arg, arginase; ADI-PEG20, pegylated arginine deiminase; BCT-100, pegylated arginase; M. hominis, *mycoplasma* hominis; M. arginine, *mycoplasma* arginine; S. pyogenes, *streptococcus* pyogenes; ASS1, argininosuccinate synthase 1; ASL, argininosuccinatelyase; OTC, ornithine transcarbamylase; HuArgI (Co)-PEG5000, pegylated human recombinant arginase I with cobalt.

Despite these advances, there is a need for a systematic review of *ex vivo* and *in vivo* studies, clinical trials, and the benefits and limitations of arginine-based therapies for glioma treatment. This review aims to summarize the current state of research on ADT and provide a basis for future investigations into this promising therapeutic approach.

## 2 Materials and methods

BOX 1 **| Search strategy for arginine deprivation therapy treating glioblastoma.**

**Pubmed (n = 337)**:

((((((ADI-PEG20(Title/Abstract)) OR (Arginine(Title/Abstract))) OR (Arg1(Title/Abstract))) OR (Arginase)) OR (canavanine)) OR (peg-bct-100)) AND (((((((((((((((((((Glioma(Title/Abstract)) OR (Gliomas(Title/Abstract))) OR (Glial Cell Tumors(Title/Abstract))) OR (Glial Cell Tumor(Title/Abstract))) OR (Tumor, Glial Cell(Title/Abstract))) OR (Tumors, Glial Cell(Title/Abstract))) OR (Mixed Glioma(Title/Abstract))) OR (Glioma, Mixed(Title/Abstract))) OR (Gliomas, Mixed(Title/Abstract))) OR (Mixed Gliomas(Title/Abstract))) OR (Malignant Glioma(Title/Abstract))) OR (Glioma, Malignant(Title/Abstract))) OR (Gliomas, Malignant(Title/Abstract))) OR (Malignant Gliomas(Title/Abstract))) OR (Glioblastoma(Title/Abstract))) OR (anaplastic astrocytoma(Title/Abstract))) OR (diffuse astrocytoma(Title/Abstract))) OR (anaplastic oligodendroglioma(Title/Abstract))) OR (oli-godendroglioma(Title/Abstract))).

**ScienceDirect (n = 374)**:

(“glioma” OR “glioblastoma”) AND (“Arginine” OR “Arginase”) NOT “target”.

**Web of Science (n = 75)**:

“glioma” OR “glioblastoma” (Title) AND “arginine” OR “arginase” (Title).

### 2.1 Literature search strategy

In accordance with PRISMA guidelines, we conducted a systematic review of the literature to identify relevant trials. Two reviewers independently searched the data using the search strategy in Box 1 to identify relevant trials published from the earliest available date to February 2024, regarding the efficacy of ADT therapy for glioblastoma. Based on Medical Subjects Headings (MeSH) indexes and keyword searches, we used a combination of terms related to the tumors – “glioma”, “glioblastoma” and “malignant glioma” - and arginine deprivation therapies – “ADI-PEG20”, “Arg1”, “arginine”, “arginase” and “PEG-BCT-100” - in PubMed, ScienceDirect and Web of Science. Data on relevant clinical trials are obtained from ClinicalTrials. As a systematic literature review does not qualify for inclusion in the PROSPERO registry, we have registered our study on the Open Science Framework (OSF) with the DOI 10.17605/OSF.IO/6J53K.

### 2.2 Eligibility criteria

Because of the scope of the review, we included trials that investigated the efficacy of arginine deprivation therapy for glioblastoma. Study types included *in vitro* experiments, *in vivo* experiments and clinical trials. All ADT-related interventions were included: ADI-PEG20, BCT-100, arginine free condition, SpyADI, HuArgI (Co)-PEG5000, and various combination therapies. The following types of articles are excluded: 1) arginine as part of the structure of other proteins; 2) protein arginine methyltransferase (PRMT) inhibitors; and 3) reviews or meta-analyses. 4) Full text not available.

### 2.3 Article screening

**FIGURE 2:**
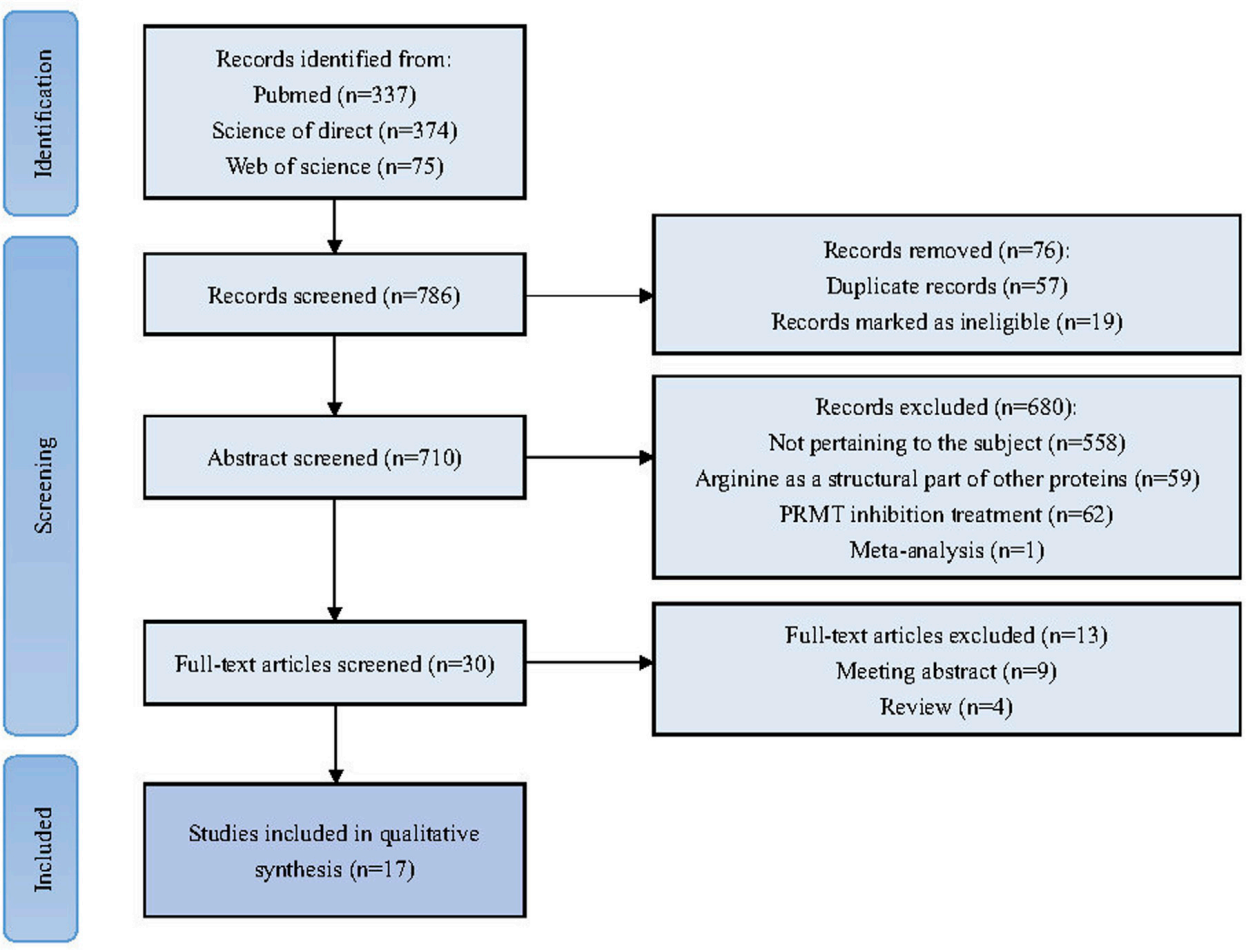
Flowchart illustrating the systematic process of conducting a literature review and meta-analysis in accordance with the PRISM guidelines.

The reviewers initiated the selection process by identifying and eliminating evidently duplicates. Subsequently, a preliminary screening was conducted based on titles and abstracts, applying the exclusion criteria to exclude any that did not meet the pre-established requirements. Following the preliminary exclusion, the full-text articles were subjected to a thorough and stringent secondary review. This detailed evaluation aimed to confirm the alignment of each study with the specific inclusion criteria set forth for this systematic review. Any discrepancies in the assessment were addressed through collaborative discussions among the reviewers, ensuring a consensus was reached on the final selection of studies for inclusion. The PRISMA flowchart for this review is shown in Figure 2.

### 2.4 Data extraction

Two reviewers extracted and organized the data. The extracted data from the studies encompassed names of authors, year of publication, materials, the experiment type, intervention details, sample size, and research conclusions. Interventions were categorized according to their modality, including Arginine free condition, ADI-related therapies, and Arg-related therapies. The ADI-related therapies included SpyADI and ADI-PEG20, while the Arg-related therapies included BCT-100 and HuArgI (Co)-PEG5000. Finally, the reviewers integrated the efficacy, mechanism, combination therapy, and clinical trial portions of the data separately and analyzed them.

## 3 Results

A total of 786 articles were collected through an initial search. 710 articles were retained after the initial screening to remove duplicates. After further screening and full text review based on the inclusion and exclusion criteria, 30 articles were selected for full analysis, of which a total of 17 were ultimately deemed relevant to the scope of this study. Tables 2, 3 summarized the list of all included studies and the data and conclusions extracted from them.

**TABLE 2:** Detailed description of the studies that used arginine deprivation therapy for treating glioma.

Author	Year	Materials	Type	Intervention	Highlights
Arginine free condition——oleh stasyk					
Iuliia Pavlyk et al.^28^	2015	Human U251-MG, U87-MG and LN229 cells	*In vitro*	Arginine free condition	Arginine, but not lysine, deprivation affected cell morphology, significantly inhibited their motility and invasiveness, and impaired adhesion. Arginine deprivation in glioblastoma induced specific changes in actin assembly, decreased β-actin filament content and affected its N-terminal arginylation
Yaroslav Bobak et al.^29^	2016	Glioblastoma U251-MG cells	*In vitro*	Arginine free condition/+canavanine	One of the responses of human solid cancer cells to arginine starvation is the induction of prolonged endoplasmic reticulum (ER) stress and activation of the unfolded protein response (UPR). The induction of ER stress and apoptosis in arginine-starved cells could be significantly enhanced by the plant-derived arginine analogue canavanine, but not by the classical ER stress inducer tunicamycin
C. Noreen Hinrichs et al.^30^	2018	The GBM cell lines U251-MG and U138-MG (both p53mut) as well as isogenic U87-MG-shLuc control cells (p53wt) and their p53-knockdown counterpart U87-MG-shp53	*In vitro*	2 U/mL yeast-derived rhArg/Arg-free culture medium	ADT by rh-Arginase inhibits 2-D GBM cell growth and recovery and radiosensitizes GBM cells, particularly in p53-deficient counterparts
Olena Karatsai et al.^31^	2020	U251-MG; U87-MG; Primary Rat Glial Cell Culture	*In vitro*	Arginine-free medium + canavanine	The combination of arginine deprivation and canavanine affected tumor cell viability, morphology, motility and adhesion
**Arginine deiminase**					
**SpyADI——Claudia Maletzki**					
Tomas Fiedler et al.^32^	2015	12 patient-derived GBM **cell** lines; subcutaneous tumor model, **mice** (HROG05)	*In vitro* and *in vivo*	SpyADI + Palomid 529/SAHA/TMZ/CQ (ADI from S. pyogenes serotype M49 strain 591 was heterologously expressed in *E. coli* Dha)	***In vitro*:** Combinations of SpyADI and Palomid 529 were most effective after 2 rounds of treatment. Comparable enhanced antitumor effects were observed after the addition of chloroquine to ADI. ***In vivo*:** ADI and SAHA combinations effectively controlled the growth of HROG05. Combinations of ADI and Palomid did not show any antitumor effect
Claudia Maletzki et al.^33^	2017	GBM cell lines (HROG05/52/63/02)	*In vitro*	Two rounds of treatment (72 h each) with SpyADI and selected drugs (2.5 μM temozolomide, 2.5 μM sorafenib, 1 nM vincristine, 1 μM curcumin, and 10 μM resveratrol	ADI first induces a cellular stress response characterized by the upregulation of genes mainly belonging to the heat shock protein family. In addition to autophagocytosis, we show for the first time that senescence is another cellular response mechanism to ADI treatment and that this bacterial enzyme can act as a radiosensitizer
Rico Schwarz et al.^34^	2022	Low-passage glioblastoma **cell** lines HROG02, HROG05, HROG52, and HROG63; **NSG mice:** SpyADI; SpyADI-PEG20; PBS	*In vitro* and *in vivo*	SpyADI vs SpyADI-PEG20	SpyADI-PEG20 demonstrated higher antitumor activity against GBM cells *in vitro* and *in vivo* than SpyADI.
Christin Riess et al.^35^	2022	Patient-derived GBM tumor cell lines and normal control cells	*In vitro*	SpyADI + CDKi (dinaciclib/abemaciclib)	All CDKi/SpyADI combinations showed synergistic antitumor effects, especially when given sequentially (SEQ)
Charlotte Linke et al.^36^	2023	Patient-derived GBM tumor cell lines: primary (HROG02, HROG52, GBM03, GBM06, GBM14, and GBM15) or recurrent (HROG05, HROG63)	*In vitro*	SpyADI + MitA	The combination of MitA and SpyADI enhanced the effect of monotherapy in 2D and 3D models by increasing cytochrome C and ATF4. The simultaneous approach was superior to sequential application
**ADI-PEG20——Nelofer Syed**					
N Syed et al.^37^	2013	22 primary GBM cultures; sixty patients (36 male, 24 female, median age 57 years)	*In vitro*	ADI-PEG20+CQ	Methylation of CpG islands of ASS1 and ASL predicts sensitivity to arginine deprivation. CQ inhibits autophagy and accelerates ADI-PEG20-induced cell death. In cells with methylated ASL, CD133 þ CSCs are inhibited by ADI-PEG20
Justyna Magdalena Przystal et al.^38^	2018	GBM **cell** lines; immunodeficient CD-1 nude **mice**	*In vitro* and *in vivo*	ADI-PEG20/+TMZ	ADI-PEG20 significantly reduces intracranial growth in ASS1-positive backgrounds. The combination of ADI and TMZ works on both positive and negative backgrounds. ADI-PEG20 can cytoreduce GBM and enhance the effects of TMZ.
Nabil Hajji et al.^39^	2022	U87 cell line and patient-derived primary GBM cell line TB48, The GL261 cells expressing GFP (GL261-GFP) and BV2 cells; GL261 mice	*In vitro* and *in vivo*	ADI-PEG20	ADI-PEG20 not only enhanced the cellular sensitivity of argininosuccinate synthetase 1-positive GBM to ionizing radiation by increasing NO production and hence the generation of cytotoxic peroxynitrites, but also promoted glioma-associated macrophage/microglial infiltration into the tumors, changing their classical anti-inflammatory (protumor) phenotype to a pro-inflammatory (anti-tumor) phenotype
**Arginase**					
**BCT-100**					
Chi Tung Choy et al.^40^	2016	GBM cell lines A172, M059, M059K, T98G, U373, U87-MG; 70 GBM patients	*In vitro*	BCT-100	BCT-100 inhibited the growth of GBM cells
**HuArgI (Co)-PEG5000**					
Oula Khoury et al.^41^	2015	GBM cell lines (U251, U87-MG, T98, U118-MG, Hs683, SF, A172, H4, SW1088) and human feta glial cells SVG-P12	*In vitro*	Human recombinant arginase I (Co)-PEG5000 [HuArgI (Co)-PEG5000] +CQ	HuArg1(Co)-PEG5000 was cytotoxic to all GBM cells, but SVG-P12 was insensitive to toxicity

Abbreviation: p53mut, p53 mutation; shLuc, short hairpin RNAs, targeting luciferase; shp53, short hairpin RNAs, targeting p53; p53 wt, p53 wildtype; rhArg, recombinant human arginase; ADT, arginine deprivation therapy; ADI, arginine deaminase; Spy (S. pyogenes), *streptococcus* pyogenes; Arg, arginase; ADI-PEG20, pegylated arginine deiminase; BCT-100, pegylated arginase; ASS1, argininosuccinate synthase 1; ASL, argininosuccinatelyase; CQ, chloroquine; NSG, mice; NOD, scid gamma mice; CDKi, cyclin-dependent kinase inhibitors; MitA, mithramycin A; CSC, cancer stem cells; GBM, glioblastoma; SAHA, suberoylanilide hydroxamic acid; TMZ, temozolomide; *E. coli* Dha, Escherishia coli DH5-Alpha; HuArg1(Co)-PEG5000, pegylated human recombinant arginase 1 with Cobalt; NO, nitric oxide.

**TABLE 3:** Current clinical trials of arginine deprivation therapy.

NCT number	Study status	Condit-ions	Inclusion	Nubmer of patients	Phases	Time frame	Interventions	Highlight
**NCT02029690**	TERMINATED	HGG	Ten 18 years of age or older patients with a histologically confirmed diagnosis of HGG >50% of tumor cells do not express ASS1 as determined by IHC	10	P1	2014/4/3	ADI-PEG20+pemetrexed + cisplatin	ADIPEMCIS was well tolerated and compared favorably with historical controls.^42^
**NCT04587830**	TERMINATED	GBM	Patients age between 20 and 75 with newly diagnosed, histologically confirmed GBM KPS≥60	23	P1	2020/9/14	ADI-PEG 20+RT + TMZ	Average peripheral blood Arg levels were suppressed in most subjects, and citrulline levels were increased in a circular fashion. The addition of ADI-PEG 20 to RT + TMX was safe and no dose-limiting toxicity was observed. Adverse reactions, still typical of RT + TMZ, increased with the addition of ADI-PEG 20.^43^
**NCT03455140**	TERMINATED	pHGG	49 HGG patients Age≤ 25	49	P1/2	2018/8/28	BCT-100	BCT-1OO was clinically safe in paediatric HGG with the best dose of 1600U/kg iv weekly.^44^
**NCT03970447**	RECRUITING	GBM	Newly diagnosed or recurrent GBM patients Age ≥18	-----	P2/3	2019/7/30	Temozolomide/Lomustine/Regorafenib/Radiation/Paxalisib/VAL-083/VT1021/Troriluzole/ADI-PEG 20	-----

Abbreviation: HGG, high grade glioma; IHC, immunohistochemistry; ASS1, argininosuccinate synthase 1; P1, phase 1; ADIPEMCIS, ADl-PEG, 20 with Pembrolizumab and cisplatin; Arg, arginase; ADI-PEG, 20, pegylated arginine deiminase; RT, radiotherapy; TMZ, temozolomide; GBM, glioblastoma; KPS, karnofsky; VAL-083, Dianhydrogalactitol; VT1021, Vigeo’s lead asset; BCT-100, pegylated arginase.

### 3.1 Therapeutic efficacy of arginine deprivation therapy

Arginine deiminase (ADI) and arginase (Arg1) have emerged as promising agents in the therapeutic armamentarium against glioma, with demonstrated anti-glioma toxicity and a reliable safety profile in both *in vitro* and *in vivo* experiments.

#### 3.1.1 Arginine deiminase (ADI)

To date, three different laboratories have independently validated the significant effects of ADI therapy on glioma cells. Oleh Stasyk’s laboratory (Pavlyk et al., 2015; Bobak et al., 2016; Hinrichs et al., 2018; Karatsai et al., 2020) used an argi-nine-free medium to simulate the effects of ADI therapy and observed a 50% reduction in cell viability at 48 h that was sustained for up to 144 h, with no adverse effects on normal glial cells (Linke et al., 2023). On the other hand, Claudia Maletzki’s laboratory (Fiedler et al., 2015; Maletzki et al., 2017; Schwarz et al., 2022; Riess et al., 2022; Linke et al., 2023), investigated the cytotoxicity of Spy ADI and observed a 50% reduction in cell viability in certain cell lines: HROG02, HROG05, HROG10 and HROG17 cells, but this was not dose dependent (Fiedler et al., 2015). PEGylation of Spy ADI was found to reduce overall activity by ap-proximately 50% and did not improve serum stability *in vitro*, but did not affect the enzyme’s affinity for arginine and significantly prolonged the arginine-depleting effect *in vivo*, enhancing antitumor activity against glioblastoma multiforme (GBM) cells (Maletzki et al., 2017). In contrast, the Nelofer Syed laboratory (Syed et al., 2013; Przystal et al., 2018; Hajji et al., 2022; Hall et al., 2019) has focused on the modified ADI-PEG20. In a mouse intracranial model, ADI-PEG20 effectively reduced peripheral blood arginine levels in as little as 48 h, significantly reduced intracranial growth and prolonged survival of GBM in ASS1-negative mice, and showed no significant toxic effects (Przystal et al., 2018).

#### 3.1.2 Arginase (Arg1)

Two studies have reported the *in vitro* efficacy of Arg1. Both BCT-100 and the addition of Co2+ showed significant *in vitro* antitumor effects. Chi Tung Choy et al. (2016) confirmed the potent *in vitro* antitumor effects of PEG-BCT-100, achieving 70%–80% growth inhibition after 48 h of treatment in various cell lines. Specifically, PEG-BCT-100 showed median effect concentration (EC_50_) values of 0.4436, 0.2979, 0.5224, 0.1333, 0.3048 and 0.3795 IU/mL against A172, M059J, M059K, T98G, U373 and U87-MG cell lines, respectively. Oula Khoury et al. (Khoury et al., 2015) found that HuArgI (Co)-PEG5000 exhibited significant toxic effects on all GBM cell lines, particularly at 48 h post-treatment, with IC50 values ranging from 152 to 736 p.m., and had no effect on normal fetal glial cells.

### 3.2 Mechanism of arginine deprivation therapy for treating glioma

**FIGURE 3:**
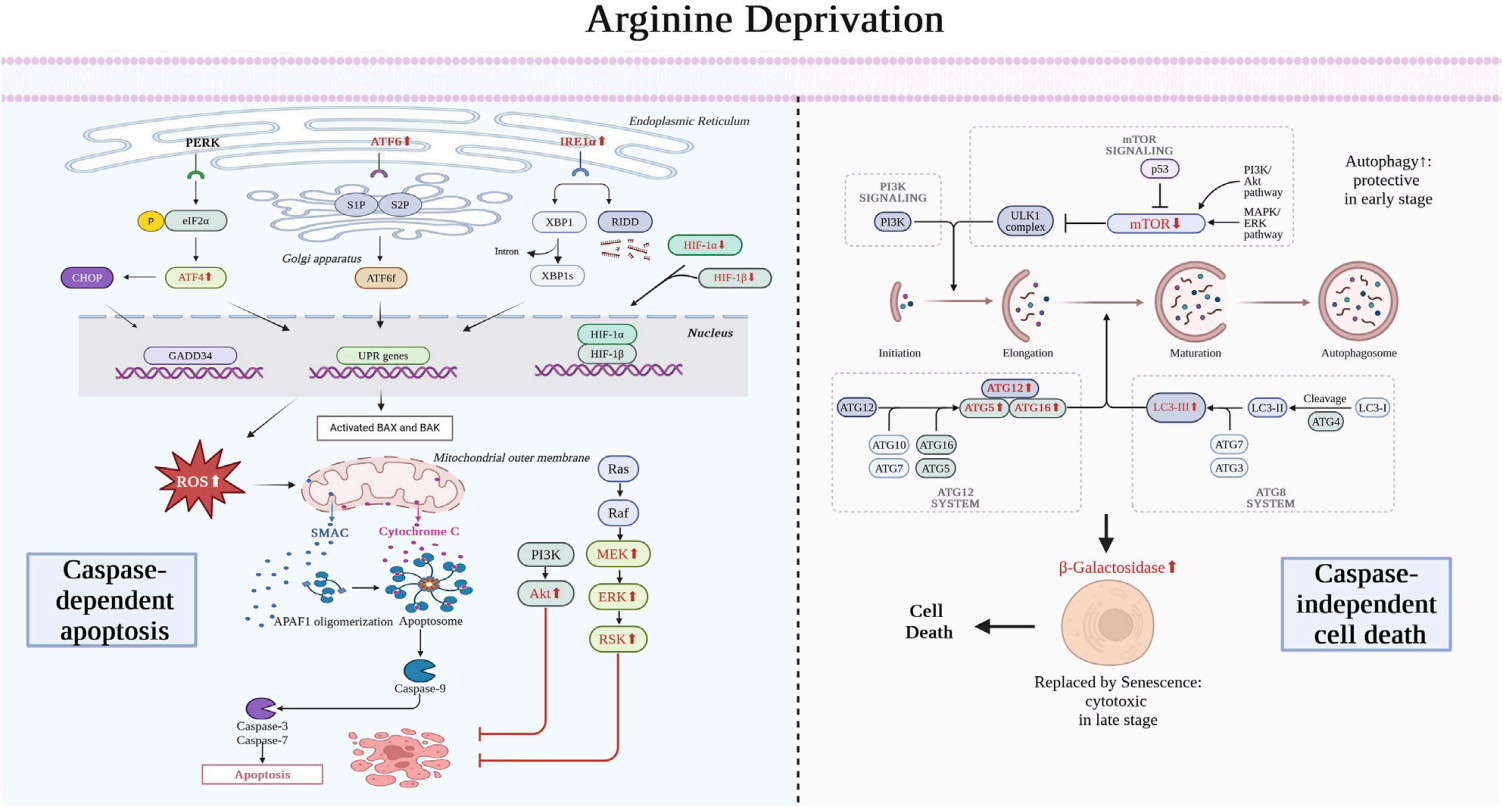
Mechanism of arginine deprivation therapy for treating glioma. In the early stages, ADT therapy leads to ER stress and autophagy in glioma cells as a protective measure, which eventually evolve into caspase-dependent apoptosis and senescence, respectively, leading to glioma cell death. The endoplasmic reticulum stress is dominated by elevated ATF6 and IRE1 expression. Causes of apoptosis also include the ROS and HIF pathways. Apoptosis arises from the release of cytochrome C from mitochondria, which ultimately leads to caspase-mediated death. However, this process is inhibited by Akt and MAPK pro-survival signaling pathways. Autophagy, on the other hand, arises from the inhibition of mTOR, which ultimately leads to the rise of ATG and LC3, which are replaced by senescence. ATF4, activating transcription factor 4; ATF6, activating transcription factor 6; eIF2α, eukaryotic translation initiation factor 2; CHOP, C/EBP-homologous protein; PERK, PKR-like ER kinase; IRE1, inositol requiring enzyme 1; XBP1, X-box binding protein 1, GADD34, growth arrest and DNA damage-inducible 34; UPR, unfolded protein response; BAX, BCL2 associated X; BAK, BCL2 antagonist/killer; HIF, Hypoxia-inducible factor; SMAC, Second Mitochondria-derived Activator of Caspases; APAF, Apoptotic Protease Activating Factor 1; Akt, Protein Kinase B; Ras, Rat Sarcoma (a family of proteins); Raf, Rapidly Accelerated Fibrosarcoma (a family of protein kinases); MEK, Mitogen-Activated Protein Kinase; ERK, Extracellular Signal-Regulated Kinase; RSK, Ribosomal S6 Kinase; PI3K, Phosphatidylinositol 3-Kinase; mTOR, Mechanistic Target of Rapamycin Kinase; ULK, Unc-51 Like Autophagy Activating Kinase; MAPK, Mitogen-Activated Protein Kinase; LC3, Microtubule Associated Protein 1 Light Chain 3; ATG, Autophagy-Related Genes; RIDD, Receptor-Interacting Protein Kinase 1 (RIPK1)-Dependent Cell Death. Created with https://www.biorender.com/, accessed on 30 July 2024.

ADT exerts an impact on GBM cells and their microenvironment through various mechanisms. In the early stages of treatment, glioma cells exhibit ER stress and autophagy as protective mechanisms, which are eventually replaced by caspase-dependent apoptosis and senescence. However, apoptosis is partially offset by pro-survival signaling pathways such as Akt and MAPK. Therefore, some studies indicate that the glioma-killing effect of ADT is mainly caspase-independent, but this view still requires further exploration. The other two causes of apoptosis are the increase in ROS and HIF. In addition, ADT can also reshape the immunosuppressive tumor microenvironment, promote the transformation of microglial phenotypes to a pro-inflammatory state, inhibit regulatory T cells (Treg), and increase the number of CD8^+^ T cells. Finally, ADT can inhibit angiogenesis, thereby affecting the survival, migration of GBM cells, and the entire microenvironment (Figure 3).

#### 3.2.1 ADT therapy inhibits growth and viability of GBM cells

The therapeutic application of ADT curbs the proliferation and survival of glioblastoma multiforme (GBM) cells. Depletion of arginine by SpyADI results in the inability of GBM cells to maintain their normal metabolic requirements, and thus a stress response will be generated to adapt to such an environment. Both cytoplasmic and endoplasmic reticulum stress-related proteins were found upregulated in SpyADI-treated GBM cells. Pro-longed stress normally leads to apoptosis, but was attenuated in this treatment due to the continued activation of pro-survival signaling pathways. In addition, GBM cells respond to stress by autophagy as an early protective mechanism, however autophagy is gradually replaced by senescence, leading to eventual cell death. Combining drugs that inhibit the protective mechanism of GBM cells against ADT therapy may be an effective way to enhance its efficacy.

SpyADI may induce cytoplasm stress. Claudia Maletzki et al. (2017) found that SpyADI-treated GBM cells showed upregulation of the heat shock proteins HSPA4/A1A/A8/A9/E1 and DNAJA4/JB12 by quantitative polymerase chain reaction (Quantitative PCR). The former is a member of the HSP70 family, which are molecular chaperones that assist in the proper folding of proteins and aid in the elimination of damaged ones, whereas the latter is part of the DnaJ family within the HSP category, acting as auxiliary chaperone proteins. These proteins recognize misfolded or unfolded proteins for refolding or degradation. Additionally, they serve to recruit and modulate the activity of Hsp70 chaperone proteins (Ryder et al., 2021). In their combined action, misfolded or unfolded proteins in the cytoplasm can be corrected to adapt to the metabolic needs of the cell.

SpyADI has been shown to induce long-term endoplasmic reticulum (ER) stress as well, which is a critical cellular response to various stressors that disrupt ER homeo-stasis (Pavlyk et al., 2015; Fiedler et al., 2015). The ER stress response is mediated by three primary ER transmembrane receptors: inositol-requiring enzyme 1 (IRE1), activating transcription factor 6 (ATF6), and protein kinase R (PKR)-like endoplasmic reticulum kinase (PERK) (Sheshadri et al., 2021). These re-ceptors play a pivotal role in the unfolded protein response (UPR), a cellular signaling pathway that aims to restore ER homeostasis and promote cell survival. Yaroslav Bobak et al. (Pavlyk et al., 2015) found *in vivo* that SpyADI induces UPR by first activating IRE1 and ATF6, leading to high expression of the ER molecular chaperone GPR94 and the ERAD-related gene EDEM1 (Flintoaca Alexandru et al., 2023; Ajoolabady et al., 2023). Although PERK is not directly activated by arginine deprivation, an increase in the phosphorylation level of eIF2α, a downstream target of PERK, leads to the upregulation of ATF4. This suggests a potential inhibition of protein synthesis as a part of the ER stress response (Gordiyenko et al., 2019). GRP94, EDEM1, and ATF4 were all upregulated in U251 cells. Subsequently, prolonged (at least up to 72 h) expression of the ER stress markers GRP78, XBP1s, and CHOP revealed the onset of ER stress.

Such prolonged expression normally induces apoptosis. To verify this, researchers compared ADI with Tunicamycin (Pavlyk et al., 2015), an ER stress inducer, however no significant phenomenon was observed in AdI. It was found that Tm causes a significant arrest of the G1/G0 cell cycle in cancer cells, whereas ADI is only slightly altered. Apoptosis was monitored by cleavage of Caspase three substrate polymerase protein 1 (PARP1) and PARP1 cleavage was found to be more pronounced with Tm treatment. That is, although SpyADI triggers persistent ER stress, its effects on cell cycle distribution and apoptosis induction are much less pronounced compared to the ER stress inducer Tm. And these are attributed to the sustained activation (up to 24 h) of Akt and p38 MAPK proteins, which mediate pro-survival signaling pathways that help cancer cells resist apoptotic signals. Cells that were more sensitive to SpyADI exhibited enhanced transcription of ATF6, ATF4, and EDEM1 genes as well as splicing of XBP1 mRNA, which is also a factor in the development of the stress response (Pavlyk et al., 2015). Given these findings, it is hypothesized that combinatorial therapies incorporating ER stress-enhancing drugs may potentiate the efficacy of ADI. Future research should focus on elucidating the interplay between the upregulation of stress-related proteins and drug resistance. Additionally, the ex-ploration of combination therapies, including the use of stress-enhancing agents or in-hibitors of pro-survival signaling pathways, in *ex vivo* models may provide valuable insights for optimizing ADI-based therapeutic strategies.

In addition, GBM cells produce autophagy. Arginine is metabolically broken down into ornithine, ammonium ions and carbon dioxide to produce energy in the form of ATP (Pols et al., 2021). Therefore, ADI leads to a decrease in ATP levels, and mTOR as a sensor of ATP is inhibited as a result (Dennis et al., 2001). This would act to enhance autophagy (Tibbo et al., 2022). In the early stages this may act as a protective mechanism (Khoury et al., 2015) to help the cell cope with stresses induced by arginine deficiency in order to delay the onset of cell death (Kim et al., 2009). It was found (Fiedler et al., 2015) that SpyADI treated GBM cells showed upregulation of Atg5, Atg12 (Jounai et al., 2007), and LC3A/B (Miao et al., 2022) cleavage, which are all autophagy-associated proteins, confirming the oc-currence of autophagy.

Senescence is a complex cellular response characterized by the irreversible loss of proliferative capacity in response to various stressors, without immediate induction of cell death. A hallmark of this state is the lysosomal hydrolase β-galactosidase (SA-β-Gal), which is selectively expressed in senescent cells and is instrumental in the hydrolysis of β-galactosides (Gasek et al., 2022). An increase in the activity of β-galactosidase was observed in SpyADI-treated GBM cells by staining the cells for β-galactosidase, which suggests that SpyADI caused senescence to occur in the GBM (Fiedler et al., 2015). Furthermore, a subset of these cells exhibited classical morphological features associated with senes-cence, including an enlargement in cell size, a change in cell morphology, and a pro-nounced cell cycle arrest. These observations provide valuable insights into the cellular mechanisms underlying the anti-proliferative effects of SpyADI and highlight the po-tential role of senescence in mediating the therapeutic response in GBM. Further in-vestigation into the molecular pathways leading to senescence and their interplay with cell cycle regulation and apoptosis may offer novel therapeutic targets for the treatment of GBM.

Long-term ADI treatment did not reveal the development of drug resistance, suggesting that ADI treatment may have a long-lasting antitumor effect (Fiedler et al., 2015). The pos-sible reason might be with increasing treatment duration, autophagy caused by ADI is gradually replaced by senescence, which is a more persistent mechanism of cell death. Ultimately, cells show a caspase-independent death phenomenon (Fenwick et al., 2024) that manifests itself as a complete cytotoxicity rather than cell cycle arrest (Karatsai et al., 2020).

#### 3.2.2 ADT blocks GBM cells migration

Arginine deprivation therapy (ADT) exerts a profound impact on the adhesion, invasive-ness, and cytoskeletal dynamics of glioblastoma (GBM) cells, predominantly through the modulation of β-actin arginylation (Pavlyk et al., 2015). By mass spectrometry, the investigators found arginine deprivation on the N-terminal aspartic acid residues of β-actin was sig-nificantly reduced in arginine deprivation-treated GBM cells. Arginylation, a crucial post-translational modification facilitated by arginylation transferase Ate1, is integral to the N-terminal processing of β-actin, which is essential for its functional role in cellular migration (Karakozova et al., 2006). Cell adhesion assays and assessment of cell-cell interactions revealed that cells in arginine-deprived environments have a diminished ability to form adhesive structures. And the results of the invasiveness test and Transwell migration assay also demonstrated a decrease in both abilities, respectively (Pavlyk et al., 2015). These collective results underscore the inhibitory effects of ADT on the adhesion, migration, and infiltration potential of tumor cells. The modulation of β-actin arginylation by ADT presents a promising therapeutic avenue for disrupting the metastatic cascade in GBM.

#### 3.2.3 ADT affects the immune microenvironment

ADT therapy can transform the polarity of microglia and simultaneously activate T cells. Microglia plays a very important role in the glioma immune microenvironment. By reducing arginine levels, ADI-PEG20 (Hajji et al., 2022) suppresses the expression of Arginase 1 (Arg1), an enzyme predominantly associated with anti-inflammatory responses (Hajji et al., 2022). This reduction in Arg1 expression prompts a pivotal shift in the polarization of micro-glia towards a pro-inflammatory phenotype. This phenotypic transition is instrumental in bolstering anti-tumor immune responses, which are critical for effective tumor sur-veillance and eradication. Furthermore, ADI-PEG20 (Hajji et al., 2022) enhances the expression of inducible nitric oxide synthase (iNOS) in microglia, which further promotes the phe-notypic shift while enhancing the activity and phagocytosis of microglia.

ADI-PEG20 elevates the levels of CD4^+^ and cytotoxic CD8^+^ T cells by down-regulating Arg1 (Hajji et al., 2022), while reduces immunosuppressive FoxP3+ regulatory T cells (Tregs) in the TME, which shifted the T cells from an immunosuppressive state to a more immunoreactive one. The reasons might be as follow: 1) The reduction of exoge-nous arginine may indirectly promote T cells to fulfill their demand for arginine through internal synthesis pathways. Activated human T cells, when deficient in extracellular arginine, induce the expression of LAT1 and 4F2hc, which allows the entry of citrulline into the intracellular compartment via the L-type amino acid transporter 1 (LAT1) (Werner et al., 2017), which leads to the generation of intracellular arginine via the citrulline/carbamoyl NO pathway. 2) Suppression of Treg cells reduces the secretion of immune-suppressing factors, which would allow CD4^+^ and CD8^+^ T cells to obtain conditions for easier pro-liferation and survival.

In addition, hypoxia is a common feature of most solid tumors. Tumors increase NO synthesis as well as VEGF expression by inducing the production of iNOS, thereby increasing angiogenesis and favoring their own growth and migration. This process is regulated by the transcription factor HIF (Burrows et al., 2016). ADI-PEG20 (Hajji et al., 2022) inhibits the expression of hypoxia-inducible factors (HIFs), in particular HIF-1α, thereby downregulating VEGF and ultimately reducing vasogenic edema and neovascularization (Redza-Dutordoir and Averill-Bates, 2016).

### 3.3 Sensitivity and resistance

#### 3.3.1 CpG island methylation in ASS1 and ASL determines the sensitivity of ADT

The methylation status of CpG islands within the ASS1 and ASL genes dictates the susceptibility of cells to ADT therapy. N syed et al. (Syed et al., 2013) reported an important finding that the level of methylation of CpG islands in ASS1 and ASL, rather than their absolute expression levels, is a key de-terminant of the sensitivity to arginine deprivation therapy. The research revealed that 30% of ASS1 and 22% of ASL CpG islands were aberrantly methylated in GBM cells. Notably, in 14% of these cases, both ASS1 and ASL exhibited methylated CpG islands, and these cells were found to be particularly sensitive to ADI-PEG20. Furthermore, ADI-PEG20 causes upregulation of ASS1 and ASL mRNA expression in GBM cells lacking CpG island methylation. Claudia Maletzki et al. (2017) found that OTC and Arg1 genes were almost always silenced in ADI-sensitive cells, thus significantly enhancing their sensitivity to ADI.

#### 3.3.2 Tumor stemness not affects the efficacy of ADT

ADT therapy may lead to the development of drug resistance, but not as a result of cancer stem cells. Poor prognosis of GBM is associated with radiotherapy-resistant CD133+ Cancer Stem Cells (CSCs). CD133+, as a pentameric transmembrane glycoprotein, is an im-portant biomarker of CSCs. Radiotherapy promotes autophagy, which prevents apop-tosis in CD133+ cells, becoming a major cause of drug resistance (Hsin et al., 2020) It was found that in the cells of methylated ASL, the ADI-PEG20 inhibited the growth of CD133+ GBM cells with the same efficiency as CD133-, suggesting that CD133+ cells do not impede the effectiveness of ADI-PEG20 treatment *in vivo* (Syed et al., 2013).

### 3.4 Impact of ADI on other therapies

#### 3.4.1 Radiotherapy

ADT has been shown to enhance the sensitivity of gliomas to radiotherapy both *in vitro* and *in vivo* (Hinrichs et al., 2018; Maletzki et al., 2017), particularly GBM cells with loss of p53 function (Hinrichs et al., 2018). In the arginine metabolism pathway, Arg1 and iNOS are the two key enzymes that have op-posing roles in the metabolism of L-arginine. Arg1 converts L-arginine to citrulline and urea, whereas iNOS converts L-arginine to NO and citrulline (Tomé, 2021). ADT inhibits Arg1 activity by depriving it of arginine: when extracellular arginine levels are reduced, Arg1 activity, which uses it as a substrate, decreases, whereas an increase in the intracellular availability of L-arginine promotes the activity of iNOS, which in turn can increase NO production. Radiotherapy kills tumor cells by damaging their DNA and other key molecules (Liu et al., 2020), leading to an increase in intracellular reactive oxygen species (ROS) (Perillo et al., 2020), including superoxide anion radicals (O2-) (Botti et al., 2010). O2- can react with NO to produce ONOO- (Hajji et al., 2022), which is able to penetrate the cell membrane (Moller and Alleva, 2023) and enter the cell interior, causing oxidative damage to cellular components such as lipids, proteins, and DNA. This oxidative damage can lead to apoptosis or necrosis of tumor cells (Redza-Dutordoir and Averill-Bates, 2016), which en-hances the therapeutic effect of radiotherapy, independent of ASS1 expression (Hajji et al., 2022). Currently, corresponding clinical trials of ADI-PEG20 in combination with radiotherapy have been conducted (Bomalaski et al., 2022). Future experiments could focus on determining the optimal dose for the combination therapy. Whether resistance develops during treatment is also an aspect that needs to be verified.

#### 3.4.2 TMZ

TMZ is a first-line agent in glioma chemotherapy (Jia et al., 2023), but the efficacy is limited due to the adverse events and drug resistance (Kourelis et al., 2015). Combination of ADI might be a potential efficient solution. By measuring luciferase activity in real time, Justyna Magdalena Przystal et al. (Przystal et al., 2018) evaluated tumor growth and regression efficacy *in vivo* and *in vitro* after treated by the combination therapy of TMZ and ADI-PEG20, finding an obvious elevation of anti-GBM activity irrespective of ASS1 status. However, the conclusion was not in line with the results of previous studies by Tomas Fiedler (Fiedler et al., 2015) and Claudia Maletzki (Maletzki et al., 2017), which did not find a synergistic effect. The reasons for the different conclusions of the three may be due to the different GBM cell lines used by the investigators, the different forms of ADI (SpyADI and ADI-PEG20) and the different experimental designs. Therefore, more experiments are needed to investigate the efficacy of this combination therapy, and PEGylation may have an influence on the result.

#### 3.4.3 Canavanine

Canavanine, a non-protein amino acid derived from legumes (Calabretta et al., 2022), is a structural analog of L-arginine, with the main difference being that Arg contains a dimethylamino group (NH2), whereas this position in canavanine is replaced by an oxygen atom to form guanidinooxy (NHO) (Staszek et al., 2017). By RT- PCR, Annexin V/malonaldehyde iodide apoptosis assay, and cell cycle analysis, Yaroslav Bobak et al. (Bobak et al., 2016) found a great enhancement of canavanine on arginine deprivation-induced endoplasmic reticulum (ER) stress and unfolded protein response (UPR). Olena Karatsai et al. (Karatsai et al., 2020) summarized the mechanism as follow: first, canavanine can promote apoptosis by reinforcing ER stress response through inhibition of mTOR activity, activation of PERK signaling cascade, and induction of CHOP synthesis. Second, it can block the reversal effect of arginine supplementation on arginine deprivation therapy, which in turn induces the caspase-dependent apoptosis in glioma cells. In addition, canavanine can destabilize the actin cytoskeleton of GBM cells and reduce their migratory capacity, as evidenced by the loss of ciliary membranes, defects in adhesion structures, and disorganization of the nuclear fibrous layer, especially the breakage of A/C- and B-type laminin, which is likely to have a close relationship with apoptosis initiation. Notably, canavanine also affects mitochondrial morphology and AMPK signaling. These new findings suggest that combining canavanine with arginine deprivation therapy may be a promising new approach to the treatment of gliomas.

#### 3.4.4 mTOR pathway inhibitor

Aberrant activation of the PI3K/Akt/mTOR pathway is a poor prognostic factor for gliomas (Ali et al., 2022). Palomid 529, a TORC1/TORC2 inhibitor, could reduce tumor growth, tu-mor angiogenesis, and vascular permeability (Xue et al., 2008). SAHA is an inhibitor of histone deacetylase (HDAC) (Karagiannis and Rampias, 2021), an enzyme that plays a key role in the epigenetic regulation of gene expression. As a FDA-approved drug for the treatment of cutaneous T-cell lymphomas, it is currently being used in clinical trials for the treatment of GBM, either as monotherapy or in combination with radiation therapy (Zhang et al., 2019). Tomas Fiedler et al. (Fiedler et al., 2015) found that the combination of ADI with Palomid 529 showed a significant synergistic effect in certain GBM cell lines, particularly in the HROG05 and HROG10 cell lines, where cell mortality reached nearly 80% after two cycles of treatments. However, no significant effect was observed *in vivo*, whereas SAHA demonstrated significant efficacy in combination with ADI both *in vitro* and *in vivo*.

Sorafenib is a multi-kinase inhibitor (Liu et al., 2006) that interferes with tumor-specific signaling pathways such as RAF/MEK/ERK or PI3K/Akt/mTOR. Curcumin has the ability to modulate core pathways involved in GBM cell proliferation, apoptosis, cell cycle arrest, autophagy, apoptosis, oxidative stress, and cell motility (Wong et al., 2021), and can increase phosphorylation levels of ERK, p38, and c-Jun proteins, thereby decreasing proliferation of GBM stem cells (GSCs) (Gersey et al., 2017). Resveratrol, a flavonoid with unique pharmacological activities, can reduce the proliferation of GBM stem cells (GSCs) by decreasing the activation of ERK, the expression of VEGF, EGF, and the markers of stemness (e.g., CD133, OCT4, and Nestin), while activating MAPK phosphatase-1 (MKP-1), thereby inhibiting cell proliferation. Furthermore, it can also increase autophagosome formation and TP53 expression, promoting autophagy and apoptosis in cancer cells (Luís et al., 2023). In addition, the anti-malarial drug quinacrine can inhibit glioblastoma by inducing oxidative stress, impairing mitochondrial function, and activating AMP-activated protein kinase (AMPK) activity in glioblastomas, as well as inhibiting angiogenesis and growth of GBM *in vivo* and acting synergistically with temozolomide (Fu et al., 2022). In 2017, Claudia Maletzki et al. (Maletzki et al., 2017) found that combining these drugs enhances the antitumor effect of ADIs, and sequential therapy is more advantageous compared to simultaneous therapy. However, whether this is a bystander effect has not been verified.

#### 3.4.5 Cell cycle protein-dependent kinases inhibitor

Cell cycle protein-dependent kinases (CDKs) play important roles in biological processes such as cell cycle control, oncogenic transcription, and DNA damage repair (Sánchez-Martínez et al., 2019). In most cancers, CDKs may undergo constitutive activation, or endogenous inhibitor deficiency (Riess et al., 2021). In 2022, by viability and toxicity assays, clone formation assays, and tumor sphere invasion experiments, Christin Riess et al. (2022) explored the anti-glioma effects of abemaciclib, a selective CDK4/6 inhibitor, and dinaciclib, a more comprehensive CDK1/2/5/9 inhibitor, in combination with SpyADI. The combination therapy was found to effectively inhibit glioma activity and could significantly impair glioma stem cell viability, invasion and growth, which is important for drug-resistant recurrence. Among them, CDKi as the first line and SpyADI as the second line is the most effective combination. Dinaciclib combined with ADI has more significant inhibitory effects on cell viability and invasion, and can effectively activate the stress sensor GADD45 and the β-linker antagonist AXIN2, thereby interfering with DNA damage repair (Rodríguez-Jiménez et al., 2021). Whereas, abemaciclib combination treatment leads to relatively more severe mitochondrial damage, manifested as mitochondrial swelling and cristae disintegration. The therapeutic effects of CDKi with ADI *in vivo* need to be further explored in the future.

#### 3.4.6 Mithramycin A (MitA)

Mithramycin A (MitA) is a sp1 blocker (Lu et al., 2023). Sp1 is a transcription factor that regulates a variety of biological processes such as cell growth, differentiation, survival, tumor progression, and metastasis, and is highly expressed in many cancer tissues (Dauer et al., 2017). In 2023, by using Western blot and flow cytometry, Charlotte Linke et al. (Linke et al., 2023) found Mit A in combination with ADI therapy upregulates ATF4 and cyto c, promoting apoptosis in GBM cells, and synchronized is superior to sequential administration, illustrating the great potential of this organic combination of therapies based on metabolic defects and cell killing SOD is an antioxidant enzyme whose primary function is to neutralize harmful superoxide radicals produced during cellular metabolism and is a stress-associated protein (Tyagi and Shumayla Singh, 2019). SOD1 observed to be upregulation suggests that tumor cells produce a certain protective mechanism. Therefore, further research is essential to elucidate the emergence of drug resistance and to assess the protective mechanism’s efficacy in depth.

#### 3.4.7 Chloroquine

Chloroquine inhibits autophagy by disrupting autophagic lysosomes (Mauthe et al., 2018). The addition of chloroquine effectively avoids protective autophagy triggered by ADI-PEG20 and accelerates the induced death. Tomas Fiedler et al. (Fiedler et al., 2015) demonstrated that chloro-quine significantly amplified the antitumor efficacy of SpyADI *in vivo*, achieving over 60% tumor cell destruction. Additionally, Oula Khoury et al. (Khoury et al., 2015) reported that the in-corporation of chloroquine into HuArgI (Co)-PEG5000 substantially increased the sensitivity of ADI to the treatment. Future studies need to focus on validating the *in vivo* efficacy of this combined therapeutic approach.

### 3.5 Clinical trials

The ongoing recruitments include a trial of ADI-PEG20 in combination with Temozolomide, Lomustine, Regorafenib, Radiation, Paxalisib, VAL-083, VT1021, and Troriluzol for the treatment of newly diagnosed or recurrent GBM (NCT03970447), and a phase 1b/2a clinical trial conducted by Beijing Tiantan Hospital on K1 injection in combination with temozolomide and radiotherapy for patients with high-grade glioma who have failed standard treatment and newly diagnosed grade IV glioma patients. Due to the limited number of existing clinical trials and insufficient dosage experience, there may be certain challenges. Moreover, current clinical trials are mostly focused on early-stage trials, focusing on safety, and the evaluation of efficacy is yet to be known. At the same time, the small number of research subjects is also one of the reasons affecting the trial conclusions. Therefore, to cope with these difficulties, future clinical trials should consider combination therapies starting with traditional drugs or experimental drugs that have been proven effective, gradually expand the scope, and consider conducting trials in multiple locations to obtain a larger number of patients. Finally, after a certain number of early clinical trials have been reached, it is possible to consider expanding to phase three clinical trials to more directly reveal the therapeutic effects.

Currently, two combination therapies of ADI-PEG20 have been validated in Phase 1 clinical trials, demonstrating its great potential for the treatment of glioma. And BCT-100 has also completed a clinical trial in pediatric and young adult glioma patients, identifying an optimal dosing regimen. The success of these compounds in preclinical studies highlights the value of arginine deprivation therapy (ADT) in the treatment of glioma and provides a solid foundation for further clinical investigation.

The success of these agents in preclinical studies underscores the value of arginine deprivation therapy (ADT) in the treatment of glioma and provides a solid foundation for further clinical investigation. In 2019, Peter E. Hall et al. (Hall et al., 2019) conducted a phase 1 clinical trial of the efficacy of ADI-PEG20 in combination with cisplatin and pemetrexed in patients with recurrent HGG who were ASS1 deficient. Pemetrexed is an inhibitor that targets TS (thymidine synthase) and DHFR (dihydrofolate reductase), two enzymes that play a key role in folate metabolism and nucleotide biosynthesis (Kesari et al., 2023). The most notable effect was in a patient with primary IDH wild-type, MGMT promoter-unmethylated glioblastoma, with a PFS of 20.8 months and an OS of more than 24 months. A total of 10 ASS1-deficient patients participated in the trial, of which eight patients (80%) had stable disease with a PFS of 5.2 months (95% CI, 2.5–20.8) and an OS of 6.3 months (95% CI, 1.8–9.7). The most common drug-related adverse events (AEs) were consistent with the common side effects of chemotherapy, namely, myelosuppression (Anand et al., 2023), including grade 4 neutropenia and thrombocytopenia. Patients experienced no bleeding events during the study, which was generally well tolerated. In 2022 (Bomalaski et al., 2022), a phase 1B clinical trial of ADI-PEG20 in combination with TMZ and radiotherapy was reported at ASCO. 23 patients were enrolled in the trial. The study found that the mPFS for this regimen was 9.5 months, and the adverse events that occurred were typical of RT + TMZ, including fatigue (52%), constipation (39%), neutropenia (39%) and allergic reactions (52%) (She et al., 2022). Mean peripheral blood Arg levels were suppressed for 4–6 weeks in most subjects, with a gradual cyclic rise in citrulline levels. The increase in anti-ADI-PEG 20 antibodies as peripheral blood arginine levels began to rebound suggests the potential for resistance development. The recommended phase II dose (RP2D) for ADI-PEG 20 was set at 36 mg/m^2^.

The significance of ADT therapy for pediatric brain tumors has also been elucidated. Despite historical conflation of pediatric and adult gliomas under 8 years of age based on morphological classification, emerging clinical and biological data point to distinct characteristics (Jones et al., 2012). While adult glioblastomas exhibit dependency on specific pathways, the expression of arginine metabolic enzymes in pediatric brain tumors remains largely unexplored. Eleanor Bishop et al. (Bishop et al., 2022) identified arginine-assisted trophic features in pediatric high-grade and low-grade gliomas, ventricular meningiomas, and medullo-blastomas, with a high percentage of tumors in pHGG samples deficient in OTC, ASS1, and ASL, suggesting potential sensitivity to arginine depletion therapy. These tumor types also showed high expression of arginase, particularly Arg2 as the major isoenzyme, suggesting their ability to transport and utilize arginine. Nicola Fenwick et al. (Fenwick et al., 2024) validated the efficacy of BCT-100 in a phase 1/2 clinical trial for gliomas in 49 young patients globally, recommending a weekly dosage of 1600 U/kg for phase 2 trials, aligning with the RP2D for adults (Yau et al., 2015).

While the three existing clinical trials, predominantly phase 1/2, demonstrate the therapeutic potential and favorable safety profile of ADT, the limited sample size necessitates further trials to consolidate these findings. This forms the basis for future expansion into phase 2/3 clinical trials, which will be crucial in advancing the application of ADT in glioma treatment.

## 4 Discussion

Arginine deprivation therapy (ADT) targets glioma cell metabolism and has shown efficacy in inhibiting tumor growth in both *in vitro* and *in vivo* studies, along with proven safety across three clinical trials. The primary mechanisms of ADT’s direct effect on glioma inhibition include inducing long-term cellular stress, autophagy, and senescence. Additionally, ADT promotes the pro-inflammatory transformation of microglial cells and activates CD8^+^ T-cells, thereby altering the immune-suppressive microenvironment to enhance the anti-glioma response. Synergistic combinations with conventional anti-glioma treatments like radiotherapy and TMZ, as well as experimental drugs such as SAHA and CDK inhibitors, have shown significant promise *in vivo* and *in vitro*, indicating new directions for ADT development.

The three main advantages of arginine deprivation therapy (Fiedler et al., 2015) are as follow: (Davis, 2018): its low dosage minimizes toxic side effects, (Śledzińska et al., 2021), it targets all tumor subpopulations, ad-dressing intra-tumor heterogeneity, and (Ostrom et al., 2021) it acts as a sensitizer to increase the responsiveness of tumor cells to chemotherapy and radiotherapy (Hinrichs et al., 2018; Syed et al., 2013). Furthermore, ADT operates through the peripheral blood and is unaffected by the blood-brain barrier, offering a unique advantage. It employs multiple mechanisms, including the induction of cellular autophagy, the generation of ROS, and the initiation of cell cycle arrest and apoptosis, positioning it as a versatile component in combination therapies.

### 4.1 The presence of discontinuation rebound and drug resistance

Re-supplementation with arginine may reverse the efficacy of ADT depending on the expression of ASS1. Tomas Fiedler et al. (Fiedler et al., 2015) found that U251 cells, when cultured without arginine and then re-exposed to a control medium for 48 h, almost fully recovered their growth and viability to match that of control cells. In addition, under arginine deprivation, these cells failed to form lamellar pseudopods, a phenomenon that was rapidly reversed upon arginine supplementation, indicating a potential risk of re-lapse post-treatment discontinuation (Pavlyk et al., 2015). However, Oula Khoury et al. (Khoury et al., 2015) when the concentration of L-citrulline was increased to 11.4 mM, it was able to rescue six HuArgI (Co)-PEG5000-treated GBM cell lines, whereas the 3 cell lines, T98, A172, and SW1088, were not recovered. In other words, even L-citrulline at very high concentra-tions could only rescue some of the GBM cell lines. Furthermore, the capacity of L-citrulline to reverse the effects was diminished when ASS1 expression was sup-pressed using RNA interference (siRNA), highlighting the significant role of ASS1 in the cell’s recovery potential. The discrepancies in the findings may be attributed to differences in the treatment protocols and the selection of cell lines. Therefore, the re-versibility of arginine deprivation therapy warrants further investigation. It is hypoth-esized that combination therapies could mitigate this issue, and this avenue of research deserves deeper exploration.

ADT may encounter resistance in the absence of arginine or citrulline supple-mentation, particularly due to the re-expression of argininosuccinate synthase (AS) or ASS1. Tsai et al. (2012) found that ADI-PEG20 can inhibit the Ras signaling pathway and its downstream effectors, ERK and the PI3K/AKT/GSK-3β kinase cascade in melanoma cells. This inhibition leads to the activation of the same signaling cascade, resulting in the phosphorylation and stabilization of the c-Myc protein. The stabilization of c-Myc subsequently suppresses its expression through the ubiquitin-mediated protein deg-radation pathway, which in turn leads to the upregulation of AS expression. Notably, c-Myc acts as a positive regulator of ASS1, and its activation is a primary driver of ASS1 re-expression (Du et al., 2022). Furthermore, Brashears et al. (2020) have confirmed the re-expression of ASS1 *in vitro*. Using activity-based proteomics (ABPP) and phosphoproteomics analyses, they revealed that the cellular response to arginine deprivation is mediated by adaptive ERK signaling and the activation of the Myc-Max transcriptional network in ADI-PEG20-senstive leiomyosarcoma cells (SKLMS-1).

Tsai et al. (2012) identified a potential therapeutic approach for melanoma by demonstrating that inhibition of the PI3K/AKT signaling pathway with a specific in-hibitor can suppress c-Myc-induced effects and significantly enhance cell killing medi-ated by ADI. This finding was corroborated in an animal model of AS-negative melanoma, where the combination of a PI3K inhibitor and ADI-PEG20 yielded a synergistic therapeutic effect superior to either agent used in isolation.

While the impact of ADI on glioblastoma (GBM) cells does not appear to be compromised by tumor stem cells leading to drug resistance (Linke et al., 2023), other tumor types have exhibited resistance phenomena and withdrawal reflexes due to the re-expression of AS or ASS1. These factors are crucial for the success of therapeutic interventions. However, the extent to which these mechanisms operate in gliomas re-mains unclear and requires further investigation. A broader range of *in vivo* and *in vitro* experiments to elucidate these processes is in great need.

### 4.2 Different therapeutic ideas targeting arginine: deprivation or grant?

Arginine deprivation therapy (ADT) targets tumor cells that rely on exogenous arginine for survival by lowering arginine levels within the tumor microenvironment. This approach differs from other ‘Arginine-grant’ treatments, such as arginase inhibitors and arginine supplementation therapies, which manipulate arginine availability in distinct ways and are associated with different mechanisms of action and anti-glioma effects. The interplay between these therapies and ADT requires further investigation.

Arginase inhibitors, which do not hinder T-cell proliferation, enhance anti-tumor responses by targeting the arginase enzymes ARG1/2 within the tumor microenvironment (TME). ARG1/2 is highly expressed in human high-grade gliomas. ARG1 is a cytoplasmic protein, while ARG2 is mainly localized in the mitochondria (Pilanc et al., 2021), closely associated with poor prognosis through degradation of L-arginine and thus inhibiting the anti-tumor effects of various types of cells in TME antitumor effects and promotes tumor cell proliferation. Paulina Pilanc et al. (Pilanc et al., 2021) found that the arginase inhibitor OAT-1746 effectively inhibited glioma cell invasion but had weak toxic effects. However, combination with a PD-1 inhibitor significantly suppressed cell viability and increased the density of CD8^+^ cells at the tumor margin. The combination therapy downregulated immune response suppressors such as ApoE, CD69 and Cxcr4, which altered the transcriptome of CD11b+ cells to restore their anti-tumor function.

Distinct from arginine deprivation therapy, which acts on the peripheral blood, arginase inhibitors focus on the tumor immune microenvironment and have a different mechanism, from which they seem not mutually exclusive. ADT often involves PEGylation to enhance the therapy’s efficacy as a starvation agent, lowering arginine levels systemically without crossing the blood-brain barrier (BBB). This reduction in peripheral arginine induces microglial cells to adopt an anti-inflammatory M1 phenotype, which enhances the phagocytosis of glioma cells. In contrast, arginase inhibitors are capable of crossing the BBB and reduce intracellular arginine levels directly. This reduction can transform the suppressive cellular phenotype within the immune microenvironment into one that is active against tumor cells. There is a hypothesis that this approach may decrease the utilization of extracellular arginine, potentially leading to higher levels of arginine in the extracellular space. However, it is yet to be confirmed whether this would counteract the effects of ADT, as no evidence has been provided to this effect. Notably, the effectiveness of arginase inhibitors is enhanced when they are used in combination with PD-1 inhibitors. ADT can be effective as a standalone treatment and is also known to have a synergistic effect when combined with other anti-tumor therapies. The importance of combination therapy is evident in both treatment modalities, highlighting their individual research value and the need for further investigation into their relationship and potential synergies.

In contrast, arginine supplementation therapy, which provides arginine to activate T cells within the immune microenvironment, has the opposite mechanism to ADT. It has primarily been studied in the context of brain metastatic tumors (Zhang et al., 2022) and is rarely used in gliomas. Given the differences in immune cell composition between brain metastases and gliomas, arginine supplementation may not be as applicable to glioma treatment. Since ADT has shown promise in T-cell activation, it may be a more viable option for patients with GBM. The role of combination therapy is significant in both approaches, and further exploration of their relationship and individual value is war-ranted.

## 5 Conclusion

Arginine deprivation therapy based on ADI-PEG20 and BCT-100 has been demonstrated anti-glioma toxicity and safety both *in vitro* and *in vivo*. The therapeutic mechanism involves inducing autophagy, senescence, and cellular stress, culminating in non-apoptotic cell death. The efficacy of ADT is influenced by the methylation status of the ASS1 and ASL genes and can be synergistically enhanced with various agents, including autophagy inhibitors and arginine analogues, to augment the impact of radiotherapy. Preliminary validation has been achieved in Phase 1 clinical trials, underscoring the significant research potential of ADT. Expanding clinical trials to further validate ADT and investigating more effective combination therapies represent promising future research avenues. However, challenges remain, with tumor resistance to arginine deprivation agents being a primary concern. This resistance may stem from the re-expression of ASS1, the development of neutralizing antibodies against the deprivation agents, and the role of autophagy (Pavlyk et al., 2015). Additionally, the therapeutic effective-ness of ADT is constrained by the concept of paracrine trophicity in tumors, specifically, the expression levels of enzymes like ASS1 in tumor cells, which can limit the therapy’s applicability.

Therefore, more biological and cytological studies are needed to further elucidate the significance of arginine on gliomas and the immune microenvironment (Wang et al., 2024). Further clarification of whether patients with gliomas should be treated with arginine deprivation therapy or arginine supplementation therapy. To confirm the clinical applicability of ADT, larger clinical trials and longer follow-up are needed to investigate the efficacy and side effects of ADT.
